# Supplementary material for: Circular insights for rhythmic health: A Bayesian approach with stochastic diffusion for characterizing human physiological rhythms with applications to arrhythmia detection
Source: PLoS One. 2025 Jun 27;20(6):e0324741. doi: 10.1371/journal.pone.0324741 (PMC12204553; doi:10.1371/journal.pone.0324741)
Supplement: This paper’s supplementary material, contained Mathematical Preliminaries, modeling framework, etc. — These materials offer essential context and details supporting the main manuscript. (PDF) [file pone.0324741.s001.pdf]

Spplimentary Materials: Circular Insights for Rhythmic Health: A Bayesian Approach with Stochastic Diffusion for Characterizing Human Physiological Rhythms with Applications to Arrhythmia Detection

Debashis chatterjee[1], Subhrajit Saha[1], Prithwish Ghosh[2,*],

1 Department of Statistics, Siksha Bhavana (Institution of Science), Visva Bharati, Bolpur, Santiniketan, 731235, India
2 Department of Statistics, North Carolina State University ,5109, SAS Hall, 2311 Stinson Dr, Raleigh, NC 27607, USA

* pghosh4@ncsu.edu

Abstract

Human physiological systems indicate a complex interplay of periodic rhythms, such as circadian and cardiovascular cycles, which vary temporally and interact. Analyzing these cyclic behaviors requires an approach that accounts for both the circular nature of the data and the random fluctuations inherent to biological processes. This paper introduces a novel multivariate circular statistical framework for modeling and analyzing human physiological rhythms. Our methodology integrates von Mises distributions, wrapped normal distributions, and stochastic diffusion processes to model multiple physiological rhythms' temporal variability and interdependence. We employ Bayesian estimation methods to extract key parameters, such as mean phase direction and concentration, and introduce advanced hypothesis testing for circular data. We demonstrated our proposed methodology through the MIT-BIH Arrhythmia Dataset analysis, highlighting the potential for circular statistical methods to provide deeper insights into physiological rhythm dynamics and their implications for human health.

Keywords: Circular Statistics, Bayesian Inference, von Mises Distribution, Stochastic Processes, Physiological Rhythms, Multivariate Analysis, ECG Signal Analysis, Hypothesis Testing, Time Series Analysis, Temporal Variability, Nonparametric Methods, Arrhythmia Detection

1 Appendix: Mathematical Preliminaries

Many fundamental definitions, like the von Mises distribution, basic wrapped normal, and Watson's U^2 test—are classical results in standard directional statistics references such as [1], [2], [3]. Doing so, we retain the essence necessary for reproducibility while removing lengthy derivations from the main text.

The rhythmic behaviors of human physiological systems, such as circadian rhythms, cardiovascular cycles, and neural oscillations, are periodic and can be represented as angular data on the unit circle. This circular nature requires specialized statistical methods, specifically from directional statistics. This section introduces a comprehensive framework for modeling and analyzing these rhythms using modern directional statistical techniques. The goal is to provide a robust methodology to analyze rhythmic data while accounting for stochastic variability and interactions between different physiological systems.

1.1 Mathematical Representation of Rhythms

Let $\theta_i(t)$ represent the i-th physiological rhythm phase at time t. Each phase $\theta_i(t) \in [0, 2\pi)$ represents an angular value corresponding to the cycle of the rhythm. For example, for a circadian rhythm, $\theta_i(t) = 0$ might correspond to midnight, while $\theta_i(t) = \pi$ corresponds to noon.

1.2 Wrapped Normal Distribution

The wrapped normal distribution is derived by wrapping a normal distribution defined on the real line onto the unit circle. This is particularly useful when the data are modeled using a normal distribution, but periodicity must be incorporated.

Given a normal distribution $N(\mu, \sigma^2)$ with mean μ and variance σ^2, the wrapped normal distribution wraps the normal density around the unit circle. The pdf of the wrapped normal distribution is given by:

$$f(\theta_i; \mu, \sigma) = \sum_{k=-\infty}^{\infty} \frac{1}{\sigma\sqrt{2\pi}} \exp\left(-\frac{(\theta_i - \mu + 2\pi k)^2}{2\sigma^2}\right),$$

where $\theta_i \in [0, 2\pi)$ is the phase, and $\mu \in [0, 2\pi)$ is the mean phase. This distribution accounts for periodicity while retaining the normal distribution's central tendency and spread properties.

The wrapped normal distribution is particularly useful when the underlying rhythmic process is symmetric but periodic. For example, this can be applied to modeling the circadian rhythm in body temperature, where the distribution of temperatures around a 24-hour cycle may resemble a normal distribution but requires circular properties.

1.3 Circular and Wrapped Distributions for Single Rhythms

Motivation for Using Circular and Wrapped Distributions Since physiological rhythms, such as circadian or cardiac rhythms, are inherently periodic, traditional linear statistical distributions are inadequate for modeling their behavior. In these cases, we turn to circular distributions, which are defined on the interval $[0, 2\pi)$, making them suitable for data that wraps around after reaching 2π, as is the case for phase angles.

Furthermore, when modeling periodic data in higher dimensions or with dependencies across different rhythms, we use wrapped distributions, where linear distributions are "wrapped" onto a circle. These wrapped distributions provide flexibility for modeling complex dependencies in multivariate rhythmic data.

Von Mises Distribution The von Mises distribution, denoted as $\mathcal{VM}(\mu_i, \kappa_i)$, is one of the most commonly used circular distributions. It is often called the "circular normal distribution" due to its similarity to the normal distribution but defined on the circle [4]. The distribution is characterized by a mean phase $\mu_i \in [0, 2\pi)$ and a concentration parameter $\kappa_i \geq 0$. The concentration parameter κ_i determines how closely the phases are clustered around the mean phase. Specifically, the probability density function (pdf) for the von Mises distribution is given by [5]:

$$f(\theta_i; \mu_i, \kappa_i) = \frac{1}{2\pi I_0(\kappa_i)} \exp\left(\kappa_i \cos(\theta_i - \mu_i)\right),$$

where $\theta_i \in [0, 2\pi)$ is the phase angle, $I_0(\kappa_i)$ is the modified Bessel function of the first kind, and κ_i represents the inverse of the dispersion. The von Mises distribution is useful for modeling rhythms that exhibit unimodal periodic behavior, such as the regular firing of neurons or the heart's diurnal cycle.

For instance, when $\kappa_i = 0$, the von Mises distribution reduces to a uniform distribution, implying no preferred phase. As κ_i increases, the phases become more tightly concentrated around the mean μ_i.

Wrapped Multivariate Normal Distribution We often need to model the joint distribution of several phase variables for physiological processes that involve multiple interacting rhythms. The wrapped multivariate normal distribution is a natural extension of the wrapped normal distribution to multiple dimensions.

The multivariate normal distribution is defined on the real line, with a mean vector $\boldsymbol{\mu}$ and covariance matrix Σ. By wrapping this distribution onto the circle, we obtain the wrapped multivariate normal distribution, which is defined by:

$$f(\boldsymbol{\theta}; \boldsymbol{\mu}, \Sigma) = \sum_{\boldsymbol{k} \in \mathbb{Z}^p} \frac{1}{(2\pi)^{p/2} |\Sigma|^{1/2}} \exp\left(-\frac{1}{2} (\boldsymbol{\theta} - \boldsymbol{\mu} + 2\pi \boldsymbol{k})^\top \Sigma^{-1} (\boldsymbol{\theta} - \boldsymbol{\mu} + 2\pi \boldsymbol{k}) \right),$$

where $\boldsymbol{\theta}$ is the vector of phase angles, $\boldsymbol{\mu}$ is the mean vector, and Σ is the covariance matrix that captures the dependencies between different rhythms.

This distribution is particularly useful for modeling joint rhythms, such as the interaction between heart rate variability and circadian rhythms, where the joint distribution of multiple phases can reveal important physiological dependencies.

Choosing the Appropriate Distribution The choice of the distribution depends on the characteristics of the physiological rhythm being modeled:

1. The von Mises distribution is ideal for unimodal, tightly concentrated rhythms.
2. The wrapped normal distribution is useful when the underlying rhythm has a normal distribution shape but requires circularity.
3. The wrapped multivariate normal distribution is essential for modeling multiple interacting rhythms and capturing their dependencies.

Each of these distributions provides flexibility for modeling different types of periodic behavior, and the choice of distribution reflects the statistical properties of the rhythm being studied.

1.4 Multivariate Rhythms on the Torus

Many physiological rhythms exhibit interdependencies, such as phase synchrony and phase shifts, especially in systems like cardiac rhythms, circadian cycles, and brain oscillations. To model these interactions between multiple rhythms, we extend the analysis to a multivariate setting on the torus, represented as

$$\mathbb{T}^p = \mathbb{S}^1 \times \cdots \times \mathbb{S}^1 \quad \text{(p times)},$$

where each dimension corresponds to a separate rhythm. This multivariate extension is appropriate because each rhythm is periodic and can be modeled as a circular variable.

Let $\boldsymbol{\Theta}(t) = (\theta_1(t), \ldots, \theta_p(t))^\top$ represent the vector of phases for p physiological systems at time t, where each $\theta_i(t) \in [0, 2\pi)$ is the phase of the i-th rhythm.

The joint distribution of these phases can be modeled using a multivariate wrapped normal distribution, which accounts for the periodicity of each rhythm. This distribution is obtained by wrapping a multivariate normal distribution onto the p-dimensional torus $\mathbb{T}^p$. Specifically, if

$$\mathbf{X} \sim N_p(\boldsymbol{\mu}, \boldsymbol{\Sigma}),$$

then the wrapped variable

$$\mathbf{\Theta} = \mathbf{X} \mod 2\pi$$

has the multivariate wrapped normal distribution. The probability density function (pdf) of the multivariate wrapped normal distribution is given by:

$$f_{\mathbf{\Theta}}(\boldsymbol{\theta}) = \sum_{\mathbf{k}\in\mathbb{Z}^p} \frac{1}{(2\pi)^{p/2}|\mathbf{\Sigma}|^{1/2}} \exp\left(-\frac{1}{2}(\boldsymbol{\theta} - \boldsymbol{\mu} + 2\pi\mathbf{k})^\top \mathbf{\Sigma}^{-1}(\boldsymbol{\theta} - \boldsymbol{\mu} + 2\pi\mathbf{k})\right),$$

where:

1. $\boldsymbol{\theta} \in [0, 2\pi)^p$ is the vector of phase angles,
2. $\boldsymbol{\mu} \in [0, 2\pi)^p$ is the mean vector,
3. $\mathbf{\Sigma}$ is the covariance matrix that encodes the dependence structure between the rhythms,
4. $\mathbf{k} \in \mathbb{Z}^p$ is a vector of integers, and
5. $|\mathbf{\Sigma}|$ denotes the determinant of the covariance matrix $\mathbf{\Sigma}$.

The wrapping operation ensures that the phases $\theta_i(t)$ remain within the circular domain $[0, 2\pi)$, preserving the periodic nature of the data.

The multivariate wrapped normal distribution allows us to model the interdependencies between multiple physiological rhythms while respecting the circular nature of the data. The covariance matrix $\mathbf{\Sigma}$ is crucial for understanding the relationships between the rhythms. A large off-diagonal entry σ_{ij} in $\mathbf{\Sigma}$ indicates strong phase coupling between rhythms i and j, meaning that their phases are synchronized or exhibit significant interactions. Conversely, small off-diagonal entries imply that the rhythms are largely independent.

Since the wrapped normal distribution involves wrapping the multivariate normal distribution onto the torus, the usual interpretations of mean and covariance need to be adapted for circular data. However, the covariance matrix $\mathbf{\Sigma}$ still provides valuable information about the dependencies between the rhythms.

To visualize the interactions between the rhythms, we consider both the 2D and 3D cases.

2D Torus: Interaction Between Two Rhythms In the case of two interacting rhythms ($p = 2$), we can visualize the joint phase distribution using the 2D torus, which can be represented as a flat square with opposite edges identified, or more intuitively as a donut-shaped surface.

We can plot the phases $(\theta_1(t), \theta_2(t))$ as points on a 2D plane, wrapping around at 2π to reflect the periodicity. Alternatively, since each $\theta_i(t)$ is an angle, we can represent the distributions of each rhythm using polar histograms.

The overlaid histograms allow us to visually assess the phase distribution and any synchrony between the two rhythms.

3D Torus: Interaction Between Three Rhythms For three interacting rhythms ($p = 3$), the visualization extends to a 3D torus. We visualize the interactions by plotting the first two phases $(\theta_1(t), \theta_2(t))$ on a 2D plane and representing the third phase $\theta_3(t)$ using color coding.

The interaction between multiple physiological rhythms is modeled using a multivariate wrapped normal distribution, where the parameters used in the simulation are as follows:

1. **Number of rhythms:** $p = 3$ (representing three distinct physiological rhythms).
2. **Mean vector:** The mean vector $\boldsymbol{\mu}$ is given by:

$$\boldsymbol{\mu} = \begin{bmatrix} 0 \\ \frac{2\pi}{3} \\ \frac{4\pi}{3} \end{bmatrix}$$

3. **Covariance matrix:** The covariance matrix $\boldsymbol{\Sigma}$, representing the dependencies between the rhythms, is specified as:

$$\boldsymbol{\Sigma} = \begin{bmatrix} 0.5 & 0.4 & 0.25 \\ 0.4 & 0.5 & 0.3 \\ 0.25 & 0.3 & 0.5 \end{bmatrix}$$

4. **Sample size:** $N = 1000$ samples are drawn from the multivariate wrapped normal distribution.

The phases $\boldsymbol{\theta}(t) = (\theta_1(t), \theta_2(t), \theta_3(t))^\top$ were wrapped to the interval $[0, 2\pi)$. The numerical values of the samples were used to generate both the 2D polar histograms and the 3D scatter plots.

The color intensity helps identify areas where all three rhythms are synchronized.

These visualizations allow us to intuitively understand the dependencies between multiple physiological rhythms. In the 2D torus, we observe the joint phase distribution of two interacting rhythms, and phase alignment can be detected through the overlap of the histograms.

In the 3D torus, the interaction between three rhythms is more complex. The color intensity helps identify areas of phase synchrony and phase shifts. Regions where the phases are aligned might correspond to specific physiological states or responses.

These visualizations demonstrate how circular distributions can be effectively extended to multivariate contexts, preserving the periodic nature of the data.

1.5 Stochastic Temporal Variability

Physiological rhythms, such as cardiac, circadian, and neural rhythms, are subject to both deterministic and stochastic influences. These rhythms exhibit natural cycles, but environmental factors (e.g., light exposure, physical activity) and internal biological noise introduce random variability. To capture the stochastic nature of these rhythms, we use stochastic differential equations (SDEs) to model the temporal evolution of the phase of each rhythm.

Stochastic Process on the Circle The phase $\theta_i(t)$ of the i-th physiological rhythm evolves over time according to the following stochastic differential equation on the circle:

$$d\theta_i(t) = \omega_i dt + \sigma_i dB_i(t),$$

where:

1. ω_i is the intrinsic angular frequency of the rhythm, representing the deterministic rate of change of the phase,
2. σ_i is the diffusion coefficient, capturing the magnitude of random fluctuations in the rhythm, and
3. $B_i(t)$ is a standard Brownian motion (Wiener process).

Since $\theta_i(t)$ is defined modulo 2π, we ensure that the phase remains within $[0, 2\pi)$ by taking $\theta_i(t) = \theta_i(t) \mod 2\pi$.

Coupled Stochastic Differential Equations To model interactions between rhythms, we can consider coupling terms in the SDEs. For example, for two rhythms, we might write:

$$d\theta_1(t) = \omega_1 dt + K_{12} \sin(\theta_2(t) - \theta_1(t))dt + \sigma_1 dB_1(t),$$
$$d\theta_2(t) = \omega_2 dt + K_{21} \sin(\theta_1(t) - \theta_2(t))dt + \sigma_2 dB_2(t),$$

where:

1. K_{12} and K_{21} are coupling constants representing the strength of interaction between the rhythms, and
2. the sine terms introduce phase-dependent coupling, modeling how the phase of one rhythm influences the rate of change of the other's phase.

This type of coupled SDEs is related to the Kuramoto model for synchronization phenomena.

Numerical Simulation of the SDEs To simulate the evolution of the phases $\theta_i(t)$, we use the Euler-Maruyama method, a numerical technique for solving SDEs. For each time step Δt, we update the phases using:

$$\theta_i(t + \Delta t) = \theta_i(t) + \omega_i \Delta t + \sigma_i \sqrt{\Delta t} \xi_i(t),$$

where $\xi_i(t) \sim N(0, 1)$ is a standard normal random variable, and we wrap $\theta_i(t + \Delta t)$ to $[0, 2\pi)$.

We simulate the temporal evolution of the physiological rhythms is simulated using stochastic differential equations (SDEs) (refer to fig **??**). The exact numerical parameters for the SDE simulation are:

1. **Number of rhythms:** $p = 3$ (representing three distinct rhythms).
2. **Angular frequencies:** The drift terms ω_i for each rhythm are set as:

$$\omega_1 = 1.0, \quad \omega_2 = 1.2, \quad \omega_3 = 1.5$$

3. **Diffusion coefficients:** The diffusion coefficients σ_i for each rhythm are set as:

$$\sigma_1 = 0.5, \quad \sigma_2 = 0.4, \quad \sigma_3 = 0.3$$

4. **Total time period:** $T = 10$ time units.
5. **Number of time steps:** $n_{\text{steps}} = 500$, giving a time step size $\Delta t = \frac{T}{n_{\text{steps}}} = 0.02$.

The SDEs are numerically solved using the Euler-Maruyama method, with random perturbations from Brownian motion. The exact simulation follows the update rule:

$$\theta_i(t + \Delta t) = \theta_i(t) + \omega_i \Delta t + \sigma_i \sqrt{\Delta t} \xi_t,$$

where $\xi_t \sim N(0, 1)$ represents standard normal random variables. The phases are wrapped to $[0, 2\pi)$ at each time step.

The angular position corresponds to the phase $\theta_i(t)$, and time progresses outward radially from the center. The random fluctuations due to the diffusion coefficients σ_i are evident as deviations in the angular position.

Interpretation By introducing stochastic diffusion processes, we capture the inherent unpredictability in physiological rhythms while preserving their periodic nature. The random fluctuations, modeled by the Brownian motion terms, affect the evolution of the phases $\theta_i(t)$, making this model suitable for real-world biological systems that exhibit both regular cycles and random variability.

References

1. Mardia KV. Statistics of directional data. Journal of the Royal Statistical Society Series B: Statistical Methodology. 1975;37(3):349–371.

2. Jammalamadaka SR, Sengupta A. Topics in circular statistics. vol. 5. world scientific; 2001.

3. Rao AD, Girija S. Angular statistics. Chapman and Hall/CRC; 2019.

4. Ghosh P, Chatterjee D, Banerjee A, Das SS. Do Magnetic murmurs guide birds? A directional statistical investigation for influence of Earth's Magnetic field on bird navigation. Plos one. 2024;19(6):e0304279.

5. A SenGupta SRJ. Topics in Circular Statistics. Volume 5. World Scientific Publishing Co. Pte. Ltd; 2001.
