# Supplementary material for: Circular insights for rhythmic health: A Bayesian approach with stochastic diffusion for characterizing human physiological rhythms with applications to arrhythmia detection
Source: PLoS One. 2025 Jun 27;20(6):e0324741. doi: 10.1371/journal.pone.0324741 (PMC12204553; doi:10.1371/journal.pone.0324741)
Supplement: Attachment — Submitted filename: Circular_Carcadian_Rythym___PLOS_ONE__-7.pdf [file pone.0324741.s002.pdf]

Point By Point Response for: Circular Insights for Rhythmic Health: A Bayesian Approach with Stochastic Diffusion for Characterizing Human Physiological Rhythms with Applications to Arrhythmia Detection

[PONE-D-24-55262] - [EMID:6210835d5e528011]

April 3, 2025

Dear Academic Editor and PLOS ONE Editorial Team,

We would like to thank you and the reviewers for your careful reading of our manuscript (PONE-D-24-55262) and for providing such constructive feedback. We have revised the manuscript substantially in response to all comments. The following are the point-by-point responses.

1 Reviewer 1 Comments and Point-by-point Responses

Reviewer 1: There are the following comments:

General Response: We thank the reviewer for the very positive and insightful review. We are grateful. We have revised as per the insightful suggestions given.

Reviewer Comments 1 : The introduction is extremely brief. It should include a formulation of the problem to be solved and a description of the main contributions of the research in the field. The information provided in section 3 is also insufficient: measurable results of the study should be presented.

Response: Thank you for this suggestion. In the revised manuscript, we have:

(a) Expanded the Introduction to clearly state the overall problem (i.e., analyzing and detecting arrhythmias and other physiological rhythms that exhibit circular and stochastic characteristics).

(b) Highlighted the major contributions of our framework. We have enumerated how our method addresses gaps in existing analyses, mainly focusing on the Bayesian approach for circular diffusion modeling and how it can improve arrhythmia detection.

(c) Clarified measurable results: We now mention in the Introduction that we achieve improved detection metrics (accuracy, recall) for arrhythmias and provide an overview of the simulation and real-data outcomes. Also, more details have already been discussed in the discussion section, which we modified according to the suggestion.

Reviewer Comments 2 : What is the fundamental scientific novelty of this manuscript? The methods used in the manuscript are well known and were not developed by the authors.

Response: We agree that von Mises distributions and stochastic diffusion have become classical tools nowadays. However, if we may say, respectfully, probably no one combined them into a hybrid structure, taking benefit of both, as we did in this paper. Our novelty lies in:

(a) Combining multivariate circular statistics (wrapped distributions) with Bayesian inference and a stochastic diffusion model in a single pipeline specifically tailored to real ECG/arrhythmia data.

(b) Introducing advanced synchronization measures (e.g., PLV, MI) within a Bayesian circular framework to capture deterministic and random heart signal phase fluctuations.

(c) Demonstrating improved performance on the MIT-BIH arrhythmia data compared to simpler methods (Fourier-based or standard linear time series) via new accuracy metrics included in Section 7.2.

With due respect, if we may say, we are probably the foremost one to unify the techniques, which nobody has done before. We have clarified these points in the Introduction Section, the discussion of the Objective section, and the revised Discussion.

This paper contains a new paradigm of scientific approaches, in terms of novelty, with immense application potential, which we plan to explore in our future research works. We mentioned already in the paper. Nonetheless, we are thankful to the reviewer for pointing this out, as we understand the writeup of the paper previously might not have prominently highlighted the novelty convincingly, which we have done in the revised manuscript. We thank the reviewer for that. We have included the following in the manuscript:

Although the von Mises distribution and stochastic differential equations (SDEs) are well-established, the core *novelty* lies in unifying these techniques—*circular Bayesian inference plus SDE-driven diffusion*—into a single pipeline tailored for physiological data. Our framework:

(a) Preserves the *circular* geometry of phase angles while allowing for time-dependent stochastic variations in the rhythms.

(b) Exploits *Bayesian hierarchical* modeling to jointly estimate concentration parameters, mean directions, and diffusion coefficients, yielding comprehensive posterior distributions for all unknowns.

(c) Demonstrates *strong empirical performance* on arrhythmia detection, outperforming or matching prior techniques in sensitivity and specificity.

Reviewer Comments 3 : The overall framework for the proposed methodology should be presented at the beginning of the manuscript.

Response: We thank the reviewer for this suggestion, which we have done thoroughly. We have added a new "Methodological Framework Overview" subsection near the end of the Introduction to give readers a roadmap before diving into detailed theory. Moreover, we included a roadmap, as suggested by the reviewer. (TikZ-based flowchart in LaTeX that illustrates the overall framework for our proposed methodology (Bayesian circular SDE model for detecting phase anomalies in physiological signals like ECG)

Reviewer Comments 4 : Section 2 should present the gaps that this study fills compared to those considered in the literature review.

Response: Thank you for this suggestion, which we have taken very seriously. We have revised that section as per suggestion. We changed the section's name to Related Work and Gaps in the Literature, and highlighted the gaps prominently. In Section 2 , we have revised the literature review:

(a) we summarize key existing methods (linear time series, wavelet transforms, standard parametric models).

(b) we state their limitations (lack of explicit circular modeling, ignoring random fluctuations).

(c) we show how our approach addresses those gaps by unifying circular and stochastic approaches in one framework.

Reviewer Comments 5 : Are omega and sigma random functions in SDE definition in section 3?

Response: Yes, in the standard form, ω and σ are treated as random variables but time-invariant (though more complicated random effects could be introduced, and we plan that in future work). In the revised manuscript, we clarified that ω is the stochastic random variable for drift term in our base model, and σ is the random variable for diffusion coefficient. Nonetheless, we note that an extension with time-varying $\omega(t)$ and $\sigma(t)$ could be implemented, and we mention this as a future direction.

Reviewer Comments 6 : Sections 4-6 contains a lot of well-known mathematical information that can be moved to the preliminaries and can be shortened.

Response: Thank you for this suggestion. We revised that and made a preliminaries section, including them briefly with proper references. To streamline the manuscript, we have moved or condensed these well-known theoretical aspects into this single *Preliminaries* section. Readers seeking more detail can refer to the references therein. We have moved the original Preliminary section into Appendix. A brief version essential version of the Preliminaries section, suitable for the main manuscript body, with a clean and professional transition referring the reader to the appendix.

Reviewer Comments 7 : What test data is used Section 7? Why do authors demonstrate the use of simple mathematical formulas?

Response: In Section 7 of the original manuscript, we apply the proposed methodology to **Record 100** (and others) from the *MIT-BIH Arrhythmia Database*, sampled at 360 Hz. Though these formulas are relatively straightforward, we present them to ensure clarity for readers unfamiliar with Hilbert-based phase extraction. We have added short clarifications on how these phases are numerically

computed and wrapped into $[0, 2\pi)$.

With due respect, if we may say, Arrhythmia detection from ECGs often requires the instantaneous phase if we want to apply circular models. Circular statistics are not that popular among interdisciplinary medical field-based academicians who might be reading this research paper. Moreover, these *"simple"* formulas are crucial for bridging typical signal processing steps (Hilbert transform) with the circular Bayesian approach. We wanted to ensure transparency and reproducibility and ensured interdisciplinary reader's readability.

Reviewer Comments 8 : The results of testing the proposed methodology have not been compared to other methods. Are the models more accurate than the previous ones? Can they be used for making predictions? Can the authors demonstrate the quality/accuracy metrics?

Response: Thank you for this important suggestion. We have included that in the revised manuscript. Please see the Simulation section, Real Data analysis section, and section *Comparisons, Accuracy Metrics, and Predictive Capability.*

The proposed Bayesian circular stochastic differential equation (SDE) is a novel framework designed specifically for phase-based anomaly detection in ECG waveforms, a capability absent in traditional amplitude-based methods like AR and Fourier analysis. Direct comparisons with these conventional approaches are misleading because they fail to capture the circular geometry and phase shifts crucial to the SDE's analysis. While preliminary comparisons highlighted the methodological mismatch, a meaningful comparative analysis is currently hindered by the lack of existing circular statistical methodologies in ECG analysis. Nonetheless, we have done the comparison with two existing popular methods, proving our proposed methodology worthy, and depending on the nature of the data, the best.

The paper's primary contribution lies in introducing this novel approach, and future research should focus on developing appropriate benchmarks and evaluation frameworks for circular statistical ECG modeling to enable fair comparisons. Comparison with circular statistical methodology with that of linear existing methodology may be questionable because of its mismatching fundamental assumptions. Nevertheless, we have done that in the revised manuscript, showing our model performance better, in general.

Reviewer Comments 9 : The further research directions should be discussed in more detail.

Response: Thank you for this suggestion. The revised manuscript now includes a more detailed roadmap of future work, e.g., extending to other physiological signals (respiratory, neural), exploring non-stationary diffusion parameters, and investigating real-time arrhythmia prediction.

2 Reviewer 2 Comments & and Point-by-point Responses

The submitted manuscript "Circular Insights for Rhythmic Health: A Bayesian Approach with Stochastic Diffusion for Characterizing Human Physiological Rhythms with Applications to Arrhythmia Detection" introduces an innovative statistical framework for analyzing human physiological rhythms, with a particular focus on circadian and cardiovascular cycles. It presents a multivariate circular model that integrates circular statistics with stochastic diffusion processes to capture the temporal variability and interdependence of these rhythms. The approach is commendable for its creative use of wrapped distributions and stochastic processes to address the complexities of physiological dynamics. While the manuscript presents a promising and interdisciplinary approach, several areas require improvement to enhance its clarity, accessibility, and impact.

Response to Reviewer 2 General Overview: We thank the reviewer for the very positive and insightful review. We are grateful. The reviewer acknowledges the interdisciplinary nature of the approach but requests clearer comparisons, justification of distribution choices, more robust simulations, handling of missing data, improved clarity in the SDE integration with circular statistics, better figure resolution, a broader scope (other rhythms or wearable data), and expanded conclusions. We have done that in the revised manuscript.

Reviewer Comments 1: The abstract fails to clearly state the study's purpose, problem statement, key comparative results (e.g., specific improvements in detection accuracy), and significant conclusions.

Response : Thank you for this suggestion. We have rewritten and revised the Abstract.

Reviewer Comments 2: The manuscript does not provide a clear rationale for selecting the von Mises and wrapped normal distributions over alternatives like the wrapped Cauchy or Kent distributions (Section 4.2, page 6). It lacks justification based on the data's characteristics, such as unimodality or heavy tails, making it difficult to assess the appropriateness of these model choices.

Response: Thank you for this important suggestion. We have added that now, in *Motivation & Outline of Proposed Methodology* section, explaining the theoretical and empirical reasons for using von Mises, as follows:

(a) ECG phase data typically exhibit a unimodal distribution with moderate dispersion, which suits von Mises better than heavy-tailed or elliptical alternatives like Kent.

(b) The von Mises distribution is computationally simpler and has well-established Bayesian estimation techniques.

(c) Empirical testing on the MIT-BIH data showed that the von Mises distribution yields a better fit (lower circular Kullback–Leibler divergence) than wrapped Cauchy in our preliminary tests.

Reviewer Comments 3: The study does not compare the proposed Bayesian approach with existing methods for physiological rhythm analysis or arrhythmia detection, such as linear time-series analysis, Fourier transforms, or machine learning classifiers (e.g., random forests or neural networks). It lacks benchmarks, such as comparing detection accuracy (e.g., F1-score) or computational efficiency (e.g., runtime in seconds), essential for demonstrating the method's advantages. Without these comparisons, readers cannot evaluate whether the approach offers meaningful improvements in sensitivity or specificity for clinical use.

Response: Thank you for this important suggestion. In the revised manuscript, We have now included that in details. Thank you very much for the suggestion. We have included that now. We made a separate section on this. We have now introduced a comparison with two benchmarks:

(a) A standard linear time-series approach (autoregressive model).

(b) A Fourier-based arrhythmia detection approach.

(c) We evaluate them on the MIT-BIH dataset and show that our Bayesian circular framework yields improvements in metrics, depending on threshold. We also discuss how the framework can be extended to short-term predictions of arrhythmic events.

Here, it is important to emphasize that our proposed Bayesian circular stochastic differential equation (SDE) methodology represents a fundamentally novel framework tailored explicitly for circular statistical modeling of ECG waveforms, particularly emphasizing phase-based anomalies. As the reviewer correctly pointed out, conventional approaches, such as linear autoregressive (AR) models and Fourier-based frequency-domain methods, are indeed well-established within ECG signal analysis. However, these traditional models inherently operate within an amplitude-based framework and do not explicitly capture or represent circular geometry or phase anomalies intrinsic to our approach.

The metrics derived from standard methods may not adequately reflect their capability to detect the circular nature of arrhythmic events addressed by our methodology. To illustrate this point, we initially conducted preliminary comparisons with standard methods (AR and Fourier-based) on simulated datasets where abnormal beats were purposefully introduced as circular (phase) anomalies. As expected, the amplitude-focused methods performed inadequately, underscoring the fundamental methodological mismatch. To honor reviewer's suggestion, we are including the comparison in the revised manuscript. However, we argue that meaningful comparative analysis may not be feasible within the current literature framework, primarily due to the absence of comparable circular-statistical methodologies in ECG analysis.

This paper's primary novelty and contribution lie precisely in proposing the first systematic application of Bayesian circular statistical approaches, particularly the use of circular stochastic differential equations, in ECG analysis. We suggest that comparative evaluations will become much more meaningful after further developing and applying circular statistical methods specifically targeting clinical ECG datasets. Future studies should explore appropriate clinical benchmarks, standardized datasets, and tailored evaluation frameworks designed explicitly for circular statistical modeling of ECG signals. These subsequent research endeavors will then naturally facilitate rigorous and meaningful comparisons between our proposed methodology and emerging circular statistical tools.

Reviewer Comments 4: The introduction of stochastic diffusion processes (Section 4.4, page 10) lacks

clarity on how these processes integrate with the circular statistical framework. It does not explain how drift and diffusion terms in stochastic differential equations (SDEs) are parameterized or how they interact with circular distributions, leaving readers unclear about the model's mechanics. Major revision is needed to provide intuitive explanations or mathematical details.

Response: Thabnk you for this important suggestion. We have revised Section to provide a clearer explanation of the SDE approach:

- The drift ω_i influences the expected progression of the phase around the circle.
- The diffusion σ_i adds random fluctuations, and we wrap the phase in $[0, 2\pi)$ after each Euler-Maruyama step.
- We connect these updates to the circular likelihood by re-centering the phase distribution after each time step, ensuring consistency with von Mises at each discrete measurement.

The drift ω_i influences the expected progression of the phase around the circle. The diffusion σ_i adds random fluctuations, and we wrap the phase in $[0, 2\pi)$ after each Euler-Maruyama step. We connect these updates to the circular likelihood by re-centering the phase distribution after each time step, ensuring consistency with von Mises at each discrete measurement.

Reviewer Comments 5: The simulation study (Section 5) is limited in scope, as it does not explore the method's performance under varying conditions such as different sample sizes (e.g., N=100 vs. N=1000), noise levels (e.g., SNR=10dB vs. SNR=20dB), or non-stationarities. This restricts the generalizability of the findings, as physiological data often exhibit diverse characteristics in clinical settings. For instance, the study reports a mean direction estimate of mu = 0.789 (true mu = 0.785) but does not assess how this accuracy holds under noisy or incomplete data. Comprehensive simulations are required to validate the method's robustness, especially for arrhythmia detection, where data quality varies.

Response: Thank you for this important suggestion. We have included that now in the subsection "Robustness Analysis Under Varying Sample Sizes and Noise Levels." Moreover, our code includes all the details that the reviewer mentions and can be easily accessed (we have already made the code public). We have expanded to include:

- Multiple sample sizes: $N = 100, 500, 1000$
- Different noise levels: (e.g., SNR = 10 dB vs. 20 dB).
- Non-stationary scenario: Where ω changes piecewise over time.

We show how well the Bayesian estimator recovers μ and κ under each scenario, demonstrating robustness. Due to page limit constraints, a more detailed simulation exposition is restrained. However, the code and datasets have been made publicly available to facilitate verification.

Reviewer Comments 6: This work does not address how the proposed methods handle missing data or outliers, such as ECG signal gaps or artifacts, which are common in physiological recordings (e.g., due to electrode disconnection). It lacks strategies for managing these issues, such as imputation techniques or robust estimation methods, reducing the approach's practical applicability. For example, in the MIT-BIH dataset, records often contain noise or missing segments, which could affect phase extraction accuracy.

Response: Thank you for this important suggestion. We have added a subsection named " Handling Outliers and Missing Data" under simulation section. We added now in the Methodology section describing:

- A simple robust approach for outliers (phase angles that deviate drastically are truncated or assigned lower weights).
- Methods for imputing missing ECG segments (e.g., spline interpolation or bridging with the average phase) prior to computing Hilbert transforms.
- The possibility of an advanced approach using particle filtering for Bayesian updating under missing data.

Reviewer Comments 7: The manuscript does not discuss extending the proposed framework to other physiological rhythms, such as respiratory or neural signals, or different datasets like wearable device data (e.g., smartwatch ECGs). It focuses narrowly on ECG rhythms, limiting its broader impact on physiological signal analysis.

Response: Thank you for this important suggestion. We mention that in the future work section and will do so as future work.

Reviewer Comments 8: The use of low-quality raster images for figures compromises the clarity of visual data, such as phase trajectories or polar histograms. Raster formats lose resolution when scaled, making it difficult to discern details like peak concentrations or synchronization patterns. High-quality vector images (e.g., SVG) are essential for maintaining clarity, especially for complex circular data.

Response: Thank you for the suggestion. We have tried to rectify, wherever possible, regenerated important figures in high-resolution vector PDF and/or SVG format to maintain clarity. We have verified that the legends, markers, and axes are legible at typical journal column widths.

Reviewer Comments 9: The manuscript neglects to discuss limitations, such as the sensitivity of circular methods to sample size or the assumption of stationarity in stochastic diffusion models. For instance, the Bayesian estimation may be biased with small samples (e.g., N¡50), but this is not addressed. Acknowledging limitations provides transparency and guides future research, enhancing the study's credibility.

Response: Thank you for this suggestion. Wer have included that in the revised manuscript. Section ("Discussion, Limitations and Future Work") now explicitly acknowledges:

- Potential bias from small sample sizes in Bayesian estimation.
- Stationarity assumptions in the SDE approach might not always hold for real-world ECG signals with abrupt arrhythmic events.

We note that extension to more complicated non-stationary processes is feasible and left for future research.

Reviewer Comments 10: Finally, the Conclusion section should be significantly expanded to include (i) the study's numerical results, (ii) a brief mention of the proposed approach's limitations, and (iii) prospects for future work.

Response: Thank you for this suggestion. We have done that (Changed the conclusion accordingly) in the revised manuscript.

Our revisions overall include:

1. A fully reworked Abstract, Introduction, Preliminaries section, clarifying our motivation, the gaps our work addresses, and our manuscript's main contributions.

2. Extended simulations with different sample sizes and noise levels to demonstrate the robustness and accuracy of our proposed framework.

3. Comparisons of our Bayesian circular diffusion approach to other existing methods for arrhythmia detection and physiological rhythm analysis.

4. More detailed discussion about methodology choices (e.g., why von Mises vs. other distributions), added limitations, and future directions.

5. Acknowledgement section, funding section, Declaration of no competing interest, Declaration of Ethics, Author contribution has been included.

We believe these revisions strengthen the manuscript and address the points raised. Below is our point-by-point "Response to Reviewers" describing each revision we have made.

We appreciate the opportunity to improve our manuscript and look forward.

Thanking you,
Sincerely,
The authors
(Dr. Debashis Chatterjee, Mr. Subhrajit Saha & Mr. Prithwish Ghosh)
