# Supplementary material for: Circular insights for rhythmic health: A Bayesian approach with stochastic diffusion for characterizing human physiological rhythms with applications to arrhythmia detection
Source: PLoS One. 2025 Jun 27;20(6):e0324741. doi: 10.1371/journal.pone.0324741 (PMC12204553; doi:10.1371/journal.pone.0324741)
Supplement: Attachment — Submitted filename: Circular_Carcadian_Rythym___PLOS_ONE__-11.pdf [file pone.0324741.s003.pdf]

Point By Point Response for: Circular Insights for Rhythmic Health: A Bayesian Approach with Stochastic Diffusion for Characterizing Human Physiological Rhythms with Applications to Arrhythmia Detection

[PONE-D-24-55262] - [EMID:6210835d5e528011]

April 23, 2025

Dear Academic Editor and PLOS ONE Editorial Team,

We would like to thank you and the reviewers for your careful reading of our manuscript (PONE-D-24-55262) and for providing such constructive feedback. We have revised the manuscript substantially in response to all comments. The following are the point-by-point responses.

1 Reviewer 1 Comments and Point-by-point Responses

Reviewer 1 Comments

The authors responded to all of my comments. However, there are still a number of significant flaws in the manuscript. First, there is a redundancy in the presentation in terms of length. 52 pages is too long for a research paper. For a PhD thesis, as indicated by the acknowledgements, this level of detail is appropriate, but not for an article. Secondly, there are multiple duplications in the material. One stochastic differential equation appears in different places. For example, in Section 9.2, the same expression appears twice. Formulas could be numbered and referred to. Figures 17 and 28 are the same. There are numerous similar comments. Finally, the approach of using wrapped distributions and other mathematical apparatus still does not appear

to be unique. There are numerous papers in this field: 10.1016/j.bspc.2023.105549, 10.1016/j.neuroimage.2021.118050, 10.7554/elife.84602, 10.1016.j.csda.2025.108168, etc. The manuscript needs a significant revision.

Point-by-point Response to Reviewer 1 Comments

We are deeply grateful to the Reviewer for this continued feedback, which we have taken very positively and tried to improve our paper accordingly. We acknowledge the significant concerns regarding length, redundancy, and novelty. We sincerely apologize for these shortcomings and are committed to a substantial revision to address them. We condensed the manuscript, eliminated all duplications (numbering equations and ensuring unique figures), and clearly articulated the unique contributions of our approach in relation to existing literature. We made a supplementary file alongside this paper where we kept all additional information. We appreciate the reviewer's guidance and will work diligently to improve the manuscript significantly.

Elaborately, we have carefully addressed each of the additional concerns raised and made significant revisions accordingly:

1. **Length and Redundancy:**
   We acknowledge the reviewer's concern regarding the manuscript length. In response, we have substantially reduced the length by removing redundant content and consolidating explanations where appropriate. The revised manuscript is now significantly shorter and more concise, while still maintaining the integrity of the scientific contribution. We kept the additional information in the Supplement file.

2. **Duplications and Formula Referencing:**
   All identified duplications, including the repeated stochastic differential equation in Section 9.2 and the duplicated Figures 17 and 28, have been resolved. Formulas are now clearly numbered and properly cross-referenced throughout the manuscript to avoid repetition and improve clarity.

3. **Novelty and Related Work:**
   We thank the reviewer for pointing out related literature. We have now explicitly discussed these references in the revised manuscript and clarified the distinctions between our work and the cited studies.

** We have now added in the related works and gaps in **Related Work and Gaps in the Literature** section, the following, explicitly, as follows:

It is important to mention that some recent studies have advanced statistical methods for phase data in neuroscientific applications. [Norouzpour and Roberts, 2023] proposed the Fcirc statistic for multi-group comparisons of discrete Fourier estimates in steady-state evoked potentials. [Wolpert and Tallon-Baudry, 2021] evaluated four existing circular tests (e.g. Phase Opposition Sum, Watson's U^2, Circular Logistic Regression) on binary outcome data. [Dimmock et al., 2023] introduced a Bayesian model for phase coherence in EEG/MEG, capturing uncertainty in phase-locking estimates. [Ding et al., 2025] developed autoregressive models on the manifold of covariance matrices to distinguish seizure dynamics.

Distinctive features of our proposed framework:

(a) *Multivariate Circular SDEs on the Torus:* We model p interacting rhythms via coupled SDEs preserving full circular geometry in continuous time.

(b) *Wrapped Distributions in a Bayesian Hierarchy:* We embed von Mises and wrapped-normal priors in a hierarchical model, estimated by Hamiltonian Monte Carlo to yield posterior distributions for mean directions, concentrations, and diffusion parameters.

(c) *Novel Multi-Sample Circular Tests:* We extend Watson's U^2 to multi-group settings and introduce permutation-based inference on the torus, enabling rigorous hypothesis tests beyond pairwise comparisons.

(d) *Robust Data-Irregularity Handling:* We propose circular-kernel weighting for outliers and spline/phase-bridging imputation for missing segments prior to Hilbert-transform phase extraction.

These innovations collectively go beyond the predominantly pairwise or univariate frameworks in the cited literature, providing a unified, statistically coherent toolkit for multivariate circular time-series analysis in physiological and other domains.

We hope that these substantial revisions meet the reviewer's expectations and demonstrate the novelty and value of our work in a more concise and focused presentation.

2 Reviewer 2 Comments & and Point-by-point Responses

I have now reviewed the revised version of the manuscript PONE-D-24-55262R1, titled "Circular Insights for Rhythmic Health: A Bayesian Approach with Stochastic Diffusion for Characterizing Human Physiological Rhythms with Applications to Arrhythmia Detection," submitted to PLOS One, along with the authors' detailed point-by-point responses to my previous comments. I would like to commend the authors for their diligent and comprehensive approach to the revision process. The response document was clear, thoughtful, and directly addressed each point raised in my initial review. The corresponding changes implemented in the manuscript are substantial and well-executed. The revisions, including the reworked Abstract and Introduction, improved rationale for methodological choices, inclusion of benchmark comparisons, expanded simulation studies demonstrating robustness, clarification of the SDE framework, strategies for handling missing data/outliers, enhanced figure quality, and a more thorough discussion of limitations and future scope, have significantly strengthened the manuscript. I am satisfied that all my previous concerns have been fully and effectively addressed. The manuscript is now clearer, more robust, and presents a more compelling case for the proposed methodology.

Therefore, I am pleased to recommend that this manuscript, in its current form, is suitable for publication in PLOS One.

Response: Thank you so much for this very positive appreciation, recommendation, and consideration. We are grateful.

We appreciate the opportunity to improve our manuscript and look forward.

Thanking you,
Sincerely,
The authors
(Dr. Debashis Chatterjee, Mr. Subhrajit Saha & Mr. Prithwish Ghosh)

References

[Dimmock et al., 2023] Dimmock, S., O'Donnell, C., and Houghton, C. (2023). Bayesian analysis of phase data in eeg and meg. *eLife*, 12:e84602.

[Ding et al., 2025] Ding, T., Nye, T. M., and Wang, Y. (2025). Manifold-valued models for analysis of eeg time series data. *Computational Statistics & Data Analysis*, 209:108168.

[Norouzpour and Roberts, 2023] Norouzpour, A. and Roberts, T. L. (2023). Fcirc statistic for steady-state evoked potentials; a generalized version of Tcirc2 statistic. *Biomedical Signal Processing and Control*, 87:105549.

[Wolpert and Tallon-Baudry, 2021] Wolpert, N. and Tallon-Baudry, C. (2021). Coupling between the phase of a neural oscillation or bodily rhythm with behavior: Evaluation of different statistical procedures. *NeuroImage*, 236:118050.
